# Dr. Rufus Somerby

**Published:** 1858-04

**Authors:** J. A. McClelland

**Affiliations:** Secretary.


Many of our readers, no doubt, have already heard of the death of Dr. RUFUS SOMERBY, late of Louisville, Kentucky, who, for more than thirty years, occupied a prominent place in the dental profession. He died on the morning of the 26th of January last, in the 63d year of his age. Immediately on the announcement of this sad event, the dentists of Louisville held a meeting, and testified their regret for the departed and their sympathy for the bereaved family of the deceased. The following proceedings of the meeting were forwarded to us for publication in the Journal. EDS.

At a meeting of the Dental Profession of Louisville, Kentucky, on the 26th January, 1858, at the residence of Dr. Samuel Griffith, called upon receiving intelligence of the death of Dr. Rufus Somerby, Dr. N. Clute was called to the chair and Dr. J. A. McClelland appointed Secretary.

The following preamble and resolutions were unanimously adopted:

It having pleased an all-wise Providence to call from the scene of his earthly labors and the field of his usefulness our friend and brother, Dr. Rufus Somerby, so widely known and appreciated for his talents, his rare professional skill, and his many admirable qualities of heart—therefore,

*Resolved*, That we have received with profound regret the intelligence of the decease of our late associate.

*Resolved*, That in the death of Dr. Somerby our profession has lost a bright ornament, dental science a zealous, intelligent advocate, and our city an excellent and useful citizen.

*Resolved*, That, with heartfelt gratitude for the services rendered by Dr. Somerby to the profession of which he was a distinguished member and to the community in which he lived, we will cherish his memory with affectionate regard.

*Resolved*, That we tender to the bereaved family of the deceased our warmest sympathy, and that the secretary be directed to transmit to them a copy of these resolutions, and furnish a copy to each of the dental periodicals and to the daily papers of the city for publication.

J. A. MCCLELLAND, *Secretary*.

